# Physiological characteristics of the extreme thermophile *Caldicellulosiruptor saccharolyticus*: an efficient hydrogen cell factory

**DOI:** 10.1186/1475-2859-9-89

**Published:** 2010-11-22

**Authors:** Karin Willquist, Ahmad A Zeidan, Ed WJ van Niel

**Affiliations:** 1 Applied Microbiology, Lund University, Getingevägen 60, SE-222 41 Lund, Sweden

## Abstract

Global concerns about climate changes and their association with the use of fossil fuels have accelerated research on biological fuel production. Biological hydrogen production from hemicellulose-containing waste is considered one of the promising avenues. A major economical issue for such a process, however, is the low substrate conversion efficiency. Interestingly, the extreme thermophilic bacterium *Caldicellulosiruptor saccharolyticus *can produce hydrogen from carbohydrate-rich substrates at yields close to the theoretical maximum of the dark fermentation process (i.e., 4 mol H_2_/mol hexose). The organism is able to ferment an array of mono-, di- and polysaccharides, and is relatively tolerant to high partial hydrogen pressures, making it a promising candidate for exploitation in a biohydrogen process. The behaviour of this Gram-positive bacterium bears all hallmarks of being adapted to an environment sparse in free sugars, which is further reflected in its low volumetric hydrogen productivity and low osmotolerance. These two properties need to be improved by at least a factor of 10 and 5, respectively, for a cost-effective industrial process. In this review, the physiological characteristics of *C. saccharolyticus *are analyzed in view of the requirements for an efficient hydrogen cell factory. A special emphasis is put on the tight regulation of hydrogen production in *C. saccharolyticus *by both redox and energy metabolism. Suggestions for strategies to overcome the current challenges facing the potential use of the organism in hydrogen production are also discussed.

## 1. Introduction

Anthropogenic CO_2 _emissions have generally been recognized as the major contributor to global warming and associated climate changes. Therefore, several measures are being taken to decrease the CO_2 _emission. In recent years, much effort has been devoted to rendering biofuel production economically competitive to that of fossil fuels, since this will contribute significantly to the reduction of energy-linked environmental impacts. In this quest, the choice of the raw material is of central concern. First-generation biofuels are produced from sucrose and starch-rich substrates, which may compete with human consumption - inevitably driving up market prices. As a remedy, more focus should be directed to second-generation biofuels, produced from non-edible lignocellulosic materials, the most naturally abundant raw material [1], as well as domestic and industrial wastes. The accompanying significant cost reductions should make biofuels more competitive.

Biohydrogen is a typical example of an environmentally friendly biofuel, with no CO_2 _emission resulting from its combustion. It can be produced from both lignocellulosic and waste materials [2-5], through biological conversion processes, such as dark fermentation and photofermentation. In the latter, biohydrogen can be produced using purple sulphur or non-sulphur bacteria that convert organic acids to H_2 _in photon-driven reactions [6,7]. Although a combination of these two processes is an interesting approach for maximum conversion of the energy contained in carbohydrate-rich substrates into H_2 _[8], only dark fermentative H_2 _production is covered in this review.

In total, 12 H_2 _molecules can be obtained per mole of glucose, based on the overall number of electrons that can be generated in the complete oxidation of the latter. However, dark fermentation is limited to a maximum H_2_-production efficiency of 33%, i.e., maximally four molecules of H_2 _can be acquired per molecule of glucose with acetate and CO_2 _as the other fermentation end products [9]. Yet, this is only possible when the H_2 _partial pressure (*P*_H2_) is kept adequately low [10], e.g. by continuous stripping of the produced H_2 _with an inert gas. However, for a cost-effective dark fermentation process it is vital to obtain significantly high H_2 _yields at relatively elevated *P*_H2_, due to the high impact of central costs of feedstock and gas upgrading [11]. Generally, mesophilic (co-)cultures reach H_2 _yields of ≤ 2 moles/mol hexose [12], thus exemplifying conversion efficiencies of merely 17%. In addition, these yields are obtained at low *P*_H2 _only [6]. On the other hand, based on thermodynamic aspects, thermophilic bacteria and archaea may produce up to the theoretical maximum of 4 mol H_2_/mol hexose [13]. In general, the low H_2 _yields obtained in practice by different organisms, in addition to the requirement for low *P*_H2_, are major obstacles that need to be overcome before biohydrogen production can be industrially feasible [6].

*Caldicellulosiruptor saccharolyticus *is an extreme thermophilic bacterium that can produce high H_2 _yields [14,15], and at the same time is relatively insensitive to high *P*_H2 _[16]. This organism has recently gained increased interest due to its ability to produce thermostable cellulolytic and xylanolytic enzymes [17-21], to grow on complex lignocellulosic carbon sources [2,22,23], and to co-metabolize a wide spectrum of monosaccharides including both pentose and hexose sugars [24]. Although *C. saccharolyticus *possesses these desirable traits and several research articles have been recently published on this heterotrophic strict anaerobe, it has not yet been the subject of a review article. Herein, the physiological properties of *C. saccharolyticus *will be analyzed to unravel the characteristics required for a superior H_2 _cell factory. To further underline current insights in this topic, the physiology of *C. saccharolyticus *will be compared with that of other H_2_-producing microorganisms.

## 2. Characteristics and natural habitat of *C. saccharolyticus*

*C. saccharolyticus *is a strictly anaerobic, extreme-thermophilic, Gram-positive bacterium that belongs to the Clostridia class. There are to date 13 characterized species in the genus *Caldicellulosiruptor*, of which *C. saccharolyticus *is described best, including its whole-genome sequence [25]. *C. bescii*, formerly known as *Anaerocellum thermophilum*, is the other species in the genus with its genome also fully sequenced [26,27]. *C. saccharolyticus *was isolated from a piece of wood in a hot spring in the Rotorua-Taupo thermal area in New Zealand, of which the waters have neutral pH, and are low in sulphate, salt content and free sugars [28]. Instead, The majority of the sugars present in this environment, such as glucose and xylose, are contained in complex (hemi)cellulosic polymers, which are difficult to access.

*C. saccharolyticus *is well adapted to the selective pressure of this habitat through the production of various hydrolyzing enzymes, possession of a wide range of high-affinity transport systems, and absence of glucose-based catabolite repression [24,25,29], which are all attractive properties for using the organism for industrial purposes. However, being sensitive to high osmotic pressures [30,31] is one of the undesirable consequences of adaptation to such environment. Interestingly, *C. saccharolyticus *is non-motile and non-sporulating [32], although it possesses a complete set of genes for flagella biosynthesis and several genes required for sporulation [25], signifying a historical genetic record of characteristics that could have been required in past habitats.

## 3. Genome analysis and carbohydrate utilisation by *C. saccharolyticus*

The genome of *C. saccharolyticus *has been sequenced and a microarray-based protocol for genome-wide transcriptome analysis has been developed, which is a major convenience for studying and understanding its physiology [25]. *C. saccharolyticus *has a relatively small genome of 2,970 kb and 2,760 protein-coding open reading frames (ORFs). From the genome-curation project, several new insights were gained such as the unusually high number of transposons and transposable derivatives *C. saccharolyticus *possesses, as compared with other organisms [25]. Since transposition increases the chance of genetic alterations, a large number of transposons can enhance the adaptability of this organism and may widen the spectrum of environmental conditions it can grow in, which is beneficial from an industrial point of view. On the other hand, this may also have negative repercussions since it can jeopardize stable fermentation processes. How to balance both impacts in practice needs to be further investigated.

A general favourable property of members of the Clostridia class is their ability to degrade complex polymers such as cellulosic and lignocellulosic materials [33]. Based on growth analysis, *C. saccharolyticus *can metabolize various carbon sources ranging from monomers, such as xylose, arabinose, glucose, fructose and galactose to α- and β-linked di- and polysaccharides, such as maltose, lactose, sucrose, starch, pullulan, threhalose, xylan and cellulose [32]. *C. saccharolyticus *can also grow and produce H_2 _from complex lignocellulosic materials, both pre-treated, such as Miscanthus hydrolysate [2], sugar beet juice [34] and paper sludge [23], and untreated, such as wheat straw [35], pine wood [22] and bagasse [35]. The fermentation of these raw materials by *C. saccharolyticus *has yielded H_2_, CO_2 _and acetate as the main metabolic end products [2,23,34,35], with a maximum H_2 _yield per consumed hexose of 3.0-3.8 mol/mol [2,34,35]. Interestingly, cultivations on Miscanthus hydrolysate were accompanied by complete depletion of oligosaccharides and simultaneous oligosaccharide and monosaccharide consumption [2]. *C. saccharolyticus *degrades complex polymers by producing several cellulolytic and xylanolytic enzymes [21]. Properties of the cellulase and hemicellulase enzymes of this organism as well as their gene structures have been extensively studied [21]. In contrast to cellulolytic clostridia [33], *C. saccharolyticus *does not contain a cellulosome complex. *C. saccharolyticus *is unusual with respect to its possession of a multifunctional, multi-domain organisation for the majority of its β-glucanases [29]. The majority of the xylanases in *C. saccharolyticus *are clustered in a large gene cluster, and another cluster of genes involved in xylose metabolism is in close proximity [21]. Detailed information on glycoside hydrolases and other carbohydrate active enzymes encoded by *C. saccharolyticus *genome can be retrieved from the Carbohydrate Active Enzymes database (CAZy; http://www.cazy.org/; [36]).

The manual curation of the genome sequence of *C. saccharolyticus *indicated that it possesses an unusually high number of ATP-binding cassette (ABC) transporter genes (i.e., at least 177 ABC transporter genes) [25]; many of which are responsible for the transport of monomeric and dimeric sugars. *C. saccharolyticus *has only one sugar phosphotransferase system (PTS), which is believed to be used for fructose transport [24,25]. When *C. saccharolyticus *was cultivated on a mixture of monosaccharides, they were consumed simultaneously but at different rates, i.e., fructose > arabinose > xylose > mannose > glucose > galactose. Unlike the case with disaccharides, the ABC transporters were not substrate specific for these monosaccharides. Instead, the affinity for the different sugars varied, with a clear preference for pentose sugars. Overall, the genome and transcription analyses revealed that *C. saccharolyticus *is well equipped for utilizing complex carbohydrates, such as starch and lignocellulosic polysaccharides [25].

## 4. Hydrogen yields

For cost-efficient biohydrogen production, it is critical to achieve high substrate-conversion efficiencies due to the central cost of the raw material and the need to reduce the chemical oxygen demand (COD) in the waste stream [6]. Strategies to improve the overall process yield include the application of hybrid two-step systems, consisting of a dark fermentation step combined with either a light fermentation step [37], or a methanogenesis step [4,38]. In this way, the products of the first fermentation process, consisting mainly of volatile fatty acids, can be converted in the second step to either H_2 _and CO_2 _or CH_4 _and CO_2_, respectively. The pros and cons of these two strategies were evaluated in a recent review [6] and will not be treated herein. Instead, we will focus on H_2 _yields of the dark fermentation step.

### 4.1 Metabolic pathways for hydrogen production

During fermentation, H_2 _is produced by anaerobic heterotrophs as a means to reoxidize their reducing equivalents. The maximum obtainable yield in a dark fermentation process is 4 moles H_2 _per mol of hexose, the so-called Thauer limit [9]. However, many H_2 _yields reported in literature are less than 50% of the Thauer limit (Table 1).

**Table 1:** Effect of growth temperature on H2 yield.

Organism	Opt T °C	Cultivation	Substrate	Maximum yield	Reference
**Mesophiles **					
*Escherichia coli *SR15	37	Fed-batch	Glucose	1.82	[85]
*Enterobacter aerogenes*	40	Batch	Glucose	1.0	[99]
*Enterobacter cloacae*	37	Packed bed	Starch	1.4	[100]
*Clostridium butyricum*	30	continuous	Glucose	2	[101]
*Clostridium acetobutilicum*	34	Fed-batch	Glucose	2	[102]
**Thermophiles**					
*Clostridium thermocellum*	60	Continuous		3.5	[103]
**Extreme thermophiles**					
*Thermotoga neopolitana*	77	Batch	Glucose	3.9	[104]
*Thermotoga elfii*	65	Batch	Glucose	3.3	[15]
*Caldicellulosiruptor saccharolyticus*	70	Continuous	Glucose	3.6	[14]
*Caldicellulosiruptor owensensis*	70	Batch	Glucose	4	[66]
*Thermoanaerobacter tengcongensis*	75	Batch	Glucose	4	[45]
*Thermoanaerobacter pseudoethanolicus*	70	Continuous	Xylose	1.2	[105]
**Hyper thermophiles**					
*Thermotoga maritima*	80	Batch	Glucose	4	[60]
*Pyrococcus furiosus*	100	Batch	Glucose	3.5	[57]
*Thermococcales kodakaraensis*	88	Continuous	Starch	3.3	[106]

A selection of mesophilic, thermophilic and extreme thermophilic H_2_-producing strains, showing their optimal growth temperature and H_2 _yields.

**Figure 1:**
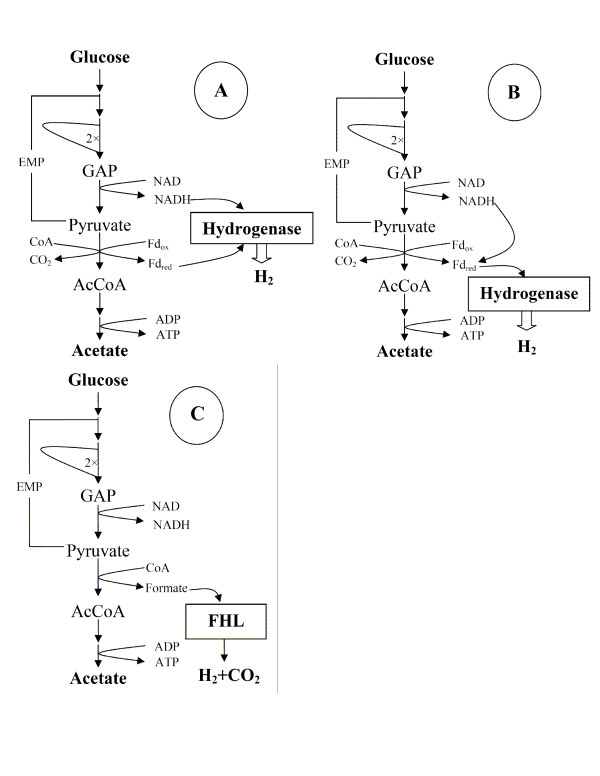
**Overview of H_2 _generation pathways in different bacteria**. In *C. saccharolyticus *(A), H_2 _is produced from NADH and Fd_red_, generated in the breakdown of glucose into acetate and CO_2 _via the EMP pathway. The H_2 _generation pathway in several clostridia carrying out acetate-type fermentation (B) is different from that in *C. saccharolyticus *in that H_2 _is generated solely from Fd_red_. NADH (generated in the EMP pathway) is converted to Fd_red _via NADH:ferredoxin oxidoreductase catalyzed reaction. In Enterobacteria (C), H_2 _is generated from formate, which is produced in the acetate pathway through the PFL-catalyzed reaction.

The maximum H_2 _yield obtained during sugar oxidation depends primarily on the catabolic pathway employed. *C. saccharolyticus*, with its H_2 _yields close to the Thauer limit [9], employs the Embden-Meyerhof-Parnas (EMP) pathway to oxidize glucose to pyruvate [14]. The genome sequence and ^13^C-NMR analysis revealed a complete gene setup for the EMP pathway and the non-oxidative part of the pentose phosphate pathway (PPP) [14,25], which enables the organism to convert different sugars into precursor metabolites and energy. No essential genes for the Entner-Doudoroff (ED) pathway have been found [14,25]. Pyruvate can be oxidized further to acetyl-CoA by pyruvate:ferredoxin oxidoreductase (PFOR), with the generation of reduced ferredoxin (Fd_red_). Molecular H_2 _can be obtained from both NADH (two moles of which are generated through the glyceraldehyde phosphate dehydrogenase (GAPDH)-catalyzed reaction in the EMP pathway) and Fd_red_, thus resulting in a maximum of 4 mol H_2_/mol hexose (Figure 1A). Several species of heterotrophic clostridia are also potentially able to produce 4 mol H_2_/mol hexose, as judged by their metabolic network (Figure 1B), when pyruvate is exclusively oxidized to acetate and CO_2_. In heterotrophic mesophilic clostridia that carry out acetate-type fermentation, NADH generated in the EMP pathway is reoxidized by NADH:Fd oxidoreductase (NFOR) with the generation of Fd_red_, which is also generated through pyruvate oxidation to acetate. The produced Fd_red _(total 8 mol/mol hexose) can subsequently donate electrons to hydrogenases that reduce protons to H_2 _[39].

On the other hand, enterobacteria are only able to produce maximally 2 moles H_2_/mol hexose, since H_2 _is generated via the hydrolysis of formate being produced from glucose through the pyruvate formate lyase-catalyzed pyruvate oxidation (Figure 1C). Interestingly, even the introduction of a synthetic ferredoxin-mediated pyruvate-to-H_2 _pathway in *E. coli *resulted in low H_2 _yields (i.e., ~2 mol/mol hexose) [40]. Some of the possible reasons why mesophilic bacteria can produce only 2 moles H_2_/mol hexose, despite being equipped with the relevant metabolic pathways, are revealed in the following sections.

### 4.2 Thermodynamic aspects

One of the reasons to which the low H_2 _yields observed in mesophilic cultures have been attributed is the thermodynamically unfavourable NADH-dependent H_2_-evolving reaction at *P*_H2 _> 0.039 kPa and at standard conditions (1 M concentration of the reactants, 25°C, pH 7) [41]. This is because H_2 _has a lower formal potential than NADH, hence for the reduction of protons by NADH to occur spontaneously, the *P*_H2 _has to be low. However, these conditions are often not encountered even when the reactor is sparged with an inert gas at a high flow rate (i.e., 400 mL/min), because the H_2 _concentration in the liquid will exceed the critical *P*_H2 _a 1000-fold [42]. Therefore, in addition to H_2_, mesophilic anaerobes normally form other reduced compounds, such as acetone, butanol, ethanol, and lactate, resulting in a H_2 _yield that usually does not exceed 2 mol/mol hexose (Table 1). A strategy to overcome this problem is to use thermophilic hydrogen producers instead, hence this works a decrease in the Gibbs free energy change of the H_2_-generation reaction in hand (Δ*G'*; [10]):

(1) $$\Delta G'=\Delta G^{0^{'}}+RT\cdot ln\frac{\left[NAD^{+}\right]\cdot P_{\text{H2}}}{\left[NADH\right]\cdot H^{+}}$$

where *∆ G*^0*' *^is the Gibbs free energy at standard conditions (kJ/mol), *R *is the gas constant (kJ/mol·K) and *T *the absolute temperature (K).

In general, extreme thermophiles reach higher H_2 _yields than mesophiles (Table 1) [13], and also at higher temperatures the critical *P*_H2 _is higher. For instance, at a temperature of 70˚C the critical *P*_H2 _is 0.173 kPa. However, this value is still well below 60 kPa, at which in practice *C. saccharolyticus *is still capable of producing 3.6-4.0 moles H_2_/mol glucose [16].

To understand this controversy, it is to be noticed that temperature is not the only factor influencing H_2 _generation. The Δ*G' *of the H_2_-producing process is also a function of the type of cofactor involved and the ratio of reduced to oxidized cofactors [43]. Each cofactor has a different formal potential, i.e., the *E*^0*' *^for Fd_red_/Fd_ox _< NADPH/NADP < NADH/NAD [36,38,39], and unless the cofactor specificity of the involved hydrogenase is known, it is not possible to determine the thermodynamics of the system. *C. saccharolyticus *possesses two distinct hydrogenases, i.e., one NADH-dependent Fe-only hydrogenase (Csac_1860-1864) and one Fd_red_-dependent, membrane-associated NiFe hydrogenase (Csac_1540-1545) [26]. Given that the formal potential of Fd_red/_Fd_ox _(*E*^0' ^approx. -400 mV) is close to that of H_2 _(*E*^0'^_H2_: -414 mV) [41], the Fd-dependent hydrogenase reaction is energetically favourable up to a *P*_H2 _of 39 kPa at standard conditions. However, this calculation is based on an approximation since the actual midpoint redox potential of Fd depends on the strain and the temperature [44]. The actual midpoint redox potential for Fd at 70ºC in *C. saccharolyticus *is not known, but should be in the same order of magnitude as found in other related thermophilic bacteria. From the thermodynamic estimation, it is obvious that it is primarily the NADH-dependent Fe-only hydrogenase that is inhibited by elevated *P*_H2_. Consistently, the NADH-dependent Fe-only hydrogenase in *Thermoanaerobacter tengcongensis *was downregulated at high *P*_H2_, while the Fd-dependent hydrogenase was constitutively expressed, independently of the *P*_H2 _[45].

Recently, Schut and Adams [46] characterized a novel type of Fe-only hydrogenase in *Thermotoga maritima *(TM1424-TM1426) that uses NADH and Fd_red _simultaneously. This novel bifurcating hydrogenase could therefore catalyze the unfavourable oxidation of NADH to H_2 _by using the exothermic oxidation of ferredoxin as a driving force. Interestingly, the Fe-only hydrogenase genes in *C. saccharolyticus *(Csac_1860, 1863-1864) show ≥ 50% sequence similarities to the bifurcating hydrogenase genes in *Tm. maritima *(TM1424-TM1426), but it remains to be investigated whether this hydrogenase in *C. saccharolyticus *possesses a bifurcating function. In fact, many of the thermophiles listed in Table 1 and several mesophilic clostridia possess a Fe-only hydrogenase-encoding genes with sequence similarity to TM1424-TM1426 in *Tm. maritima *[46]. This observation could give a plausible explanation to the ability of these thermophiles to produce high H_2 _yields in well-sparged bioreactors.

The ratio of reduced to oxidized cofactors has also a significant impact on the thermodynamics of the system (Eq. 1), which was confirmed experimentally by Velt et al [43]. In *C. saccharolyticus*, the NADH/NAD ratio changed six folds during growth-phase transition in batch cultures [47], and consequently the value of the critical *P*_H2 _for hydrogen production will also vary with the growth state of the organism. Similar observations were reported for *Clostridium cellulolyticum *[48], *Cl. butyricum *[49] and *Cl. acetobutylicum *[50]. Therefore, based on the reasoning above, the thermodynamic constraint might not be an absolute parameter for determining the critical *P*_H2 _for hydrogen production.

However, the generally higher H_2 _yields obtained by thermophilic organisms still implies that temperature has a significant impact. An intriguing question that arises is why these thermophiles have evolved for optimized H_2 _production. Resolving this phenomenon would aid in designing highly efficient H_2 _cell factories.

## 5. Effect of *P*_H2 _versus dissolved H_2 _concentration

As discussed above, due to gaps in the knowledge on the redox system in *C. saccharolyticus*, the thermodynamic constraint cannot be used to estimate its tolerance to H_2_. For practical reasons, the *P*_H2 _in the gas phase is generally used as a measure for H_2 _tolerance. The critical value of *P*_H2 _(10-20 kPa) as found for *C. saccharolyticus *is often quoted in the literature, and is defined as the partial pressure of H_2 _at which lactate formation, an alternative way for reoxidizing NADH, is initiated [30]. This critical *P*_H2 _was determined in batch cultures of *C. saccharolyticus *on sucrose using a closed bioreactor without sparging. However, in a similar experimental setup, but with xylose as the substrate, the critical *P*_H2 _was up to 60 kPa, upon which the metabolism shifted to lactate [16]. A possible explanation for the observed discrepancy can be based on the difference in the organism's metabolic activity on the two substrates, i.e., the volumetric H_2 _productivity is lower on xylose than on sucrose which results in different concentrations of dissolved H_2 _[16]. The variation in the observed critical *P*_H2 _values can also be a consequence of a different distribution pattern of catabolic and anabolic fluxes on the two substrates, leading to different metabolite levels that modulate lactate dehydrogenase (LDH) activity [47]. In that sense, the critical *P*_H2 _has no strict value, but will depend on the organism, the substrate and probably other environmental factors of the fermentation process. To make it more complicated, in batch cultures on glucose (5 g/L), lactate formation is triggered at the transition to the stationary phase, even though the *P*_H2 _was low (6.3 kPa) [47]. Therefore, there are obviously other factors that initiate lactate formation [47], making its use as a proper criterion for defining the critical *P*_H2 _questionable. Despite lactate being formed, growth still continues, but rather in a linear than an exponential fashion [16]. In addition, in continuous cultures of *C. saccharolyticus *on glucose, growth and H_2 _production were still observed at a *P*_H2 _of 67 kPa at a low dilution rate (0.05 h^-1^) [47].

Kraemer et al [51] could not find any coherent correlation between the sparging gas flow rate and H_2 _yield under different fermentation conditions. Due to this ambiguity, one may wonder whether *P*_H2 _is an appropriate parameter for inferring the critical H_2 _concentration for growth and hydrogen production. Indeed, Pauss et al [52] demonstrated in various bioreactor systems that the dissolved H_2 _easily supersaturates the liquid phase and its concentration is far from equilibrium with the gas phase. This can be attributed to the low solubility of H_2 _in water and that H_2 _production takes place in the liquid phase. The dissolved H_2 _concentration is a function of H_2 _productivity and the mass transfer rate [16], where the latter is a function of gas sparging rate and the stirring rate. A study with *C. saccharolyticus *in a pH and temperature controlled stirred tank reactor confirmed that the dissolved H2 concentration is a more indicative parameter for inhibition of H_2 _production [16]. The supersaturation of H_2 _in the liquid enacts a decline in H_2 _productivity via a metabolic shift to lactate, which may be a mechanism to steer away from exceeding the critical dissolved H_2 _concentration at which growth is inhibited [16]. To maintain a high productivity the dissolved H_2 _concentration should be kept low through increasing the mass transfer rate in the reactor, e.g. by using an appropriate sparging gas and/or proper reactor design.

Nonetheless, since data on dissolved H_2 _concentrations are scarce in the literature and *P*_H2 _is usually the determined parameter, the *P*_H2 _will continue to be used for comparisons in this review.

### 5.1 Hydrogen tolerance and sulphur reduction

Based on thermodynamics, the heterotrophic archaeon *Pyrococcus furiosus*, with its growth optimum near 100°C [53], should reach a higher H_2 _yield at higher *P*_H2 _than that obtained with *C. saccharolyticus *[10]. This organism also has an unusual advantage in that its GAPDH counterpart, glyceraldehyde-3-phosphate: ferredoxin oxidoreductase (GAPOR), generates Fd_red _[54], which has an exceptionally low formal potential (*E'*_Fd,100°C _= -600 mV; [44]). Therefore, based only on thermodynamic constraints (Eq. 1; [41]), *P. furiosus *should be the least H_2_-inhibited anaerobe listed in Table 1[41]. Yet, it produces only 2-3 moles H_2_/mol hexose, it is highly sensitive to an increase in *P*_H2 _[55], and, when present, it prefers to reduce S^0 ^instead of protons. In nature, low-*P*_H2 _environments are usually achievable for H_2 _producers living in symbiosis with hydrogenotrophic methanogens [10].

This preference for S^0 ^can be explained by examining the physiological properties of its hydrogenases, of which *P. furiosus *possesses three distinct sets. It has two cytosolic NiFe-hydrogenases, which can reduce either protons to H_2 _or polysulphide to H_2_S, using NADPH as an electron donor. It also has a membrane-bound NiFe-hydrogenase, which can reduce protons to H_2 _using Fd_red _as an electron donor [51], with the unique capability of generating a proton motive force via proton translocation [56]. Moreover, it has a NiFe-containing hydrogenase complex [51], but its physiological role is partly unknown. As a third set, the organism possesses a ferredoxin:NADP oxidoreductase (or sulphide dehydrogenase) that regulates product formation depending on the *P*_H2 _[51]. In other words, with increasing *P*_H2_, this enzyme becomes more active and decreases H_2 _production by transferring electrons from Fd_red _to either polysulphide or NADP [51]. NADPH can then be used to reduce pyruvate to alanine, catalyzed by an NADPH-dependent glutamate dehydrogenase and alanine aminotransferase [57].

S^0 ^reduction by *Thermoanaerobacter tengcongensis *enhances growth, and in its absence the organism is highly sensitive to H_2 _and growth is strongly inhibited at *P*_H2 _above 10 kPa [45]. There are, however, *Thermoanaerobacter *species that are H_2_-tolerant, such as the ethanol-adapted *T. thermosulfurigenes *[58]. This adapted strain is able to maintain growth at 1 atm H_2 _by redirecting its metabolism to ethanol, but its H_2 _yield is not higher than 50% of the Thauer limit [58].

Members of the genus *Thermotoga*, which are isolated from both shallow and deep sea hydrothermal vents rich in S^0 ^[59], are normally able to reduce either S^0 ^or thiosulphate [60]. The members of this genus display varying degrees of sensitivity to H_2_, but H_2 _inhibition can be generally relieved by the addition of S^0^, most likely as the result of a "detoxification" reaction [61]. For instance, the *Thermotoga *sp. strains FjSS3.B1 and FjSS.B1 are both highly sensitive to H_2 _and the addition of S^0 ^can re-establish their growth [61]. Although not generally required for its growth, *Tm. maritima *is able to reduce S^0^, but does not respire it, decreasing its H_2 _yield by 40% [62]. In a H_2_-pressurized reactor (H_2_/CO_2_, 80/20%; 300 kPa), however, growth of *Tm. maritima *cells was not observed unless S^0 ^was added [61]. Johnson et al [63] demonstrated that *Tm. maritima *was strongly inhibited by H_2_, which triggers the cells to enter stationary phase. Consistently, Schröder et al [64], demonstrated that *P*_H2 _levels above 2.3 kPa might be inhibitory to growth of *Tm. maritima*. However, since the organism was cultivated under uncontrolled conditions, other effects, such as the growth-associated drop in pH, might have contributed to growth inhibition. Although *Marinitoga *is closely related to *Thermotoga*, members of this genus can generally tolerate more H_2 _than *Thermotoga *species. For instance, *M. hydrogenotolerans *can grow at 1 atm H_2 _without any reduction in biomass yield, but then ethanol is formed under these conditions, which drains the electrons required for H_2 _production. In addition, in the presence of S^0^, H_2 _is not formed [65].

In contrast, members of the genus *Caldicellulosiruptor *are generally unable to reduce S^0 ^or thiosulphate [29,62,63]. Moreover, both *C. saccharolyticus *and *C. owensensis *are capable of growing and producing H_2 _at *P*_H2 _levels up to 67 and 44 kPa, respectively [16,66]. Similarly, growth of C. *kristjanssonii *was not inhibited at *P*_H2 _levels up to50 kPa [67].

The information gathered from the literature so far, in addition to our own work, reveals that S^0^-reducing thermophiles are generally more sensitive to H_2 _than thermophiles that are unable to use S^0 ^as an electron acceptor. This conclusion supports the previously proposed correlation between the ability to reduce S^0 ^and the sensitivity to H_2 _inhibition [60]. Due to inconsistencies in the experimental design for the different microorganisms studied and the lack of data on dissolved H_2 _concentrations, one should be aware that it is difficult to quantify H_2 _tolerance. In conclusion, based on this knowledge, it can be suggested to select for non-S^0^-reducing H_2_-producing candidates and future attempts to isolate promising H_2 _producers should be targeting S^0^-poor environments.

### 5.2 H_2 _removal from the liquid

To obtain higher H_2 _yields and productivities, high dissolved H_2 _concentrations should be avoided [51]. Therefore, methods to increase the mass transfer rate of H_2 _from the liquid to the gas phase should be employed. Several studies have been dedicated to develop different ways for efficient removal of H_2 _from the liquid phase (Table 2) [31].

**Table 2:** Comparison of different sparging conditions

Sparging gas	Separation	Benefits	Drawbacks	Reference
N_2_	Difficult	Not inhibitory	Difficult to separate from H_2 _Expensive	[10,28]
CO_2_	Easy	Easily separated Produced in the process	Increases osmolarity Inhibits H_2 _productivity	[10,28]
CH_4_	Easy	Easily separated Produced in a hybrid biohydrogen/biogas process	No results available for *C. saccharolyticus*	[5]
Under-pressure	Easy	Not inhibitory No dilution of the gas Cheap separation	Expensive at large scale Risk of contamination	[68]
No sparging	Easy	No dilution of the gas Cheap separation	Inhibits H_2 _productivity Inhibits H_2 _yield	[16]

Currently, N_2 _is commonly used as a sparging gas for lab-scale H_2 _production. However, applying this method at an industrial scale is not cost-effective since N_2 _is inert, and thus difficult to remove from the effluent gas stream [11]. Instead, CO_2 _could be an appropriate alternative since it can be more readily separated from H_2 _and, as a convenience, it is a product of the fermentation process itself [11]. Nevertheless, being chemically non inert, CO_2 _can influence the metabolism of some microorganisms as well as the composition of the culture medium, and hence may not be viewed as an adequate universal sparging gas. In *C. saccharolyticus *cultures, CO_2 _was already shown to negatively influence growth and H_2 _production rate [31]. In that study, higher partial pressures of CO_2 _(*P*_CO2_) were found to increase the concentration of dissolved CO_2_, which is hydrolyzed to bicarbonate and protons. This requires the addition of larger amounts of a caustic agent to compensate for the decrease in pH, contributing to an increased osmotic pressure, which inhibits growth in *C. saccharolyticus*, and a higher environmental burden [31]. Methane is another interesting alternative sparging gas that was previously shown to improve the H_2 _yield of a mesophilic consortium by 88% [5]. However, the effect of this gas on H_2 _production by *C. saccharolyticus *has not been investigated yet.

Other strategies, such as applying reduced pressure to remove H_2 _and CO_2 _from the headspace, have been shown to efficiently increase H_2 _yields in *Enterobacter cloacae *at lab scale [68]. However, on an industrial scale, this strategy might not be economically feasible due to high energy cost and increased contamination risk. Instead, two other alternative approaches may be pursued: i) improvement of reactor design to decrease the levels of dissolved H_2 _and CO_2_, and ii) metabolic engineering of the organism to enable it to either produce higher H_2 _yields at high *P*_H2 _or to withstand higher osmotic pressures.

## 6. Sugar transport and energy conservation

Based on its genome sequence and transcriptome analysis, *C. saccharolyticus *appears to rely primarily on ABC transporters for sugar uptake [23,26]. Bacterial and archaeal ABC transporters consist of five subunits, i.e., a sugar-binding protein, two transmembrane domains and two nucleotide-binding domains [69]. A common characteristic of the sugar binding protein is its ability to capture the sugar outside the cell via a cap-lock mechanism, which determines the high affinity of the transporter for the substrate. Despite the large number of ABC transporters in *C. saccharolyticus *[26], none of them have been characterized so far. However, based on transcriptional analysis, it has been concluded that, in *C. saccharolyticus*, the same transporter might translocate different sugars with varying affinities [23,26].

From a physiological perspective, ABC transporters have the disadvantage of relatively high energy demand, compared with the phosphoenol pyruvate (PEP)-dependent phosphotransferase system (PTS) (Table 3) [70]. In the latter system, one mole of the high-energy compound PEP is required for both sugar transport and subsequent phosphorylation [70], whereas two moles of ATP are required for the same steps using an ABC transporter. The fuelling of these energy demanding, high-affinity transport systems in a strict anaerobe such as *C. saccharolyticus *requires an efficient energy-conserving metabolism. It can be hypothesized that one of these energy-conserving strategies is the oxidation of glucose to acetate and CO_2_, which results in two extra moles of ATP per mole of hexose on top of the usual ATP yield of 2 mol/mol hexose in the EMP pathway. Thus, with the conversion of glucose to two moles of acetate, maximally four moles of ATP can be acquired, with the net yield being only three moles if an ABC transporter is involved (Table 3). This means that with acetate as the end product, the desired H_2 _production becomes advantageous for the microorganism since it serves as an electron sink in the re-oxidation of the reduced cofactors Fd_red _and NADH generated in the breakdown of sugars.

**Table 3:** Theoretical ATP yield (YATP/S) (mol/mol) of anaerobic glucose oxidation with different transport systems and fermentation products.

Transport	Fermentation product					
	acetate	lactate	ethanol	butyrate	butanol	propionate
ABC	3	1	1	2	1	1
PTS	4	2	2	3	2	2

To investigate the presented hypothesis further, the genomes of known H_2_-yielding anaerobes were analyzed to evaluate whether they possess PTS- or ABC-type of sugar transport. Indeed, there is a clear correlation between high H_2 _yields and the use of ABC transporters (Tables 1 &4). Organisms that were able to produce high H_2 _yields generally possess mainly ABC-type transporters, whereas low H_2_-yielding bacteria utilize more PTSs for transport. This correlation between H_2 _yields and possession of ABC transporters is more pronounced in thermophiles. For example, the extreme thermophile *Thermoanaerobacter pseudoethanolicus*, which has a PTS for glucose uptake, is unable to produce more than 2 moles H_2_/mol hexose (101), and mainly forms ethanol as a metabolic end product instead of acetate.

**Table 4:** The type of transport system with respect to growth temperature.

Organism	Opt T °C	Transporters	Reference
*Enterobacter aerogenes*	37	PTS	[107]
*Enterobacter cloacae*	37	PTS	[107]
*Clostridium butyricum*	30	PTS	This study
*Clostridium acetobutylicum*	34	PTS	[108]
*Clostridium thermocellum*	60	ABC	[109]
*C. saccharolyticus*	70	ABC	[26]
*Thermotoga neopolitana*	77	ABC	[110]
*Thermoanaerobacter tengcongensis*	75	ABC	This study
*Thermoanaerobacter pseudoethanolicus*	70	PTS	This study
*Pyrococcus furiosus*	90	ABC	This study

A selection of mesophilic, thermophilic and extreme thermophilic H_2_-producing strains, showing their optimal growth temperature, H_2 _yields and type of glucose transport. The type of glucose transport assigned to each organism was either retrieved from literature or based on bioinformatic analysis (This study) using the Integrated Microbial Genomes (IMG) database http://img.jgi.doe.gov/

### 6.1 PPi as an additional energy carrier

Another strategy to conserve energy employed by *C. saccharolyticus *is to utilize inorganic PPi as an additional energy carrier [71]. This strategy is widely applied by anaerobes and other organisms that can acquire only low energy outputs in their metabolism to obtain sufficient ATP [72], of which *Tm. maritima *[73] and various *Clostridium *species [72,73] are a few examples that have been studied to some extent.

The primary sources of PPi are poly-nucleic acid biosynthesis from (deoxy)nucleotide triphosphates and activation of amino acids and fatty acids for protein and lipid synthesis, respectively, which are reactions that operate close to equilibrium. Therefore, subsequent PPi hydrolysis is required for driving these reactions forward [74]. Moreover, studies on *E. coli *have shown that maintaining low PPi concentrations through effective PPi hydrolysis is required for rapid growth [75]. In *E. coli*, as in a majority of other organisms, PPi hydrolysis is catalyzed by a cytosolic inorganic pyrophosphatase (PPase), with the dissipation of the free-energy change of the bound PPi (Δ*G*^0'^=-21.8 kJ/mol; [76]) as heat.

**Figure 2:**
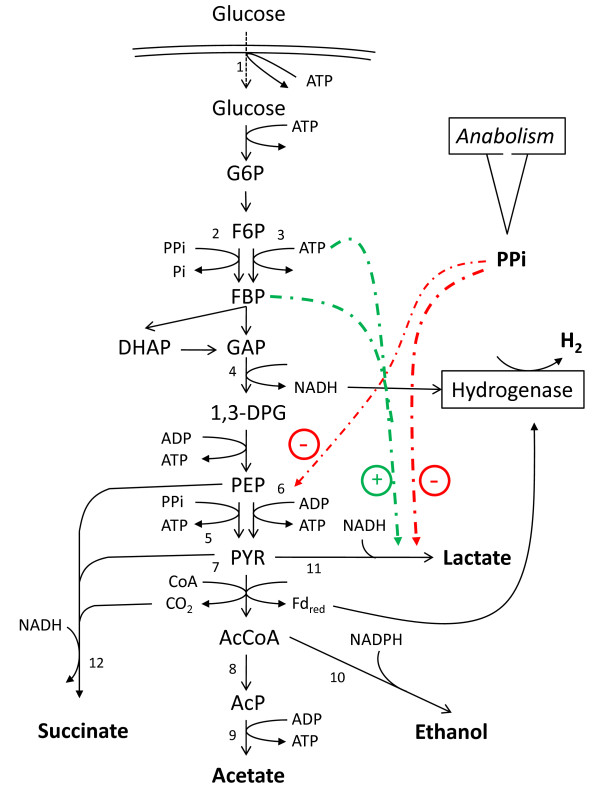
**Proposed catabolic network in *C. saccharolyticus***. Glucose is transported over the membrane by an ABC transport system (1) [23,26] and oxidized to pyruvate in the EMP pathway [13,26], with the possibility that PPi can potentially replace ATP, i.e., PPi-PFK (2) instead of ATP-PFK (3), or ADP, i.e., PPDK (5) instead of PYK (6) [71]. NADH generated through the GAPDH-catalyzed reaction (4) and Fd_red _generated from the PFOR-catalyzed reaction (7) can donate electrons for the generation of molecular H_2 _catalyzed by cytosolic NADH-dependent Fe-only hydrogenase and membrane-bound Fd-dependent NiFe-hydrogenase, respectively [26]. Acetate is formed by the consecutive actions of phosphotransacetylase (PTA; 8) and acetate kinase (AK; 9), with the generation of ATP. Alternative routes for NADH reoxidation are the formation of lactate (LDH; 11), ethanol (ADH; 10) or succinate (12) [16]. The solid lines represent metabolic routes, whereas the dashed lines represent metabolite activation (+) or inhibition (-) of enzyme activities. PPi generated from anabolic reactions [71] is a strong inhibitor of both PYK and LDH activities [47,71].

*C. saccharolyticus *lacks a cytosolic PPase. Instead, the energy of PPi hydrolysis can be conserved, for instance through: (i) an active membrane-bound PPase employing the free-energy change of the phosphate bond hydrolysis to establish a proton motive force that could be utilized for transport of nutrients or for ATP synthesis; (ii) a PPi-dependent phosphofructokinase (PPi-PFK) in addition to the ATP-dependent one (ATP-PFK); and (iii) a PPi-dependent pyruvate phosphate dikinase (PPdK) in addition to the ADP-dependent pyruvate kinase (PYK; Figure 2) [71].

Interestingly, there are indications that the PPi-level follows the growth dynamics of *C. saccharolyticus*. When the cells were growing exponentially at the maximum growth rate, the PPi levels were relatively high (4 ± 2 mM), and when the growth rate decreased, the PPi levels declined concordantly. The dynamics in PPi levels were shown to be associated with the low cytosolic PPase activity in *C. saccharolyticus *[71]. Consistently, the PPi levels followed the same growth-dependent trend in *Moorella thermoacetica *and *Cl. pasteurianum*, both of which also lack a cytosolic PPase [76]. In contrast, in *E. coli *possessing elevated cytosolic PPase activity (0.54 U/mg cdw), the PPi levels were low (0.3 mM) and did not fluctuate during growth [76].

Unlike PPi, the ATP levels in *C. saccharolyticus *were very low (0.43 ± 0.07 mM) during the exponential growth phase, but increased 2 folds at the beginning of the stationary phase [71]. Interestingly, the ADP concentration was higher than that of ATP during both phases [71], which is generally considered to be a sign of starvation. Similar low ATP/ADP ratios were observed in *Cl. acetobutylicum *[77]. Nevertheless, *C. saccharolyticus *grew exponentially, suggesting that it relies on other energy carriers, such as PPi, that may contribute to its energy charge in addition to ATP [71].

**Figure 3:**
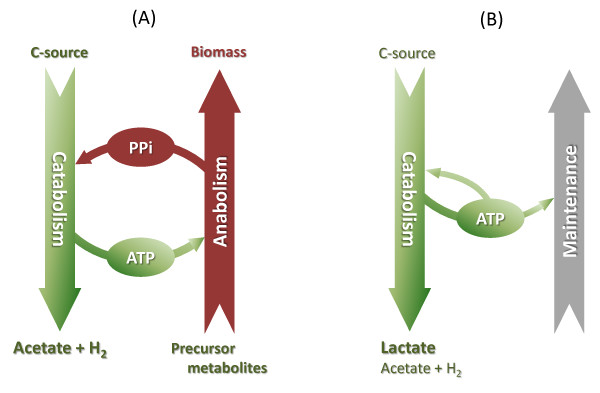
**Schematic representation of the suggested circulation of energy carriers in catabolism and anabolism in *C. saccharolyticus *during exponential growth (A), and non-growth (B), and its influence on metabolite formation**. During exponential growth, there is a high energy demand in the cells. The formation of acetate allows maximum ATP gain and high levels of PPi secure economical use of ATP, including the inhibition of LDH activity and prevention of lactate formation. During non-growth, the ATP demands in the cells are reduced and LDH activity is restored due to the absence of PPi.

The use of PPi as an additional energy carrier also introduces greater metabolic flexibility. In addition, it creates a stronger link between anabolism and catabolism, i.e., the net ATP generated in catabolic pathways is used as an energy input in biosynthetic pathways, which generates PPi that can participate in driving catabolism and generating ATP (Figure 3). In this cycle, both ATP and PPi may be key regulators of catabolic and anabolic fluxes.

### 6.2 Enzyme regulation

Regulation of the classical glycolysis and the flux distribution at the pyruvate node is a very complex and highly tuned process, since it is the principal provider of energy carriers and redox equivalents that need to be precisely balanced under all growth conditions. Regulation takes place at metabolite level as well as at enzyme and gene expression levels. Enzyme activities normally are regulated via direct mass action law (concentrations of substrates or effectors) or via allosteric inhibition or activation [78]. To maintain high growth rate conditions, cells of *C. saccharolyticus *should obtain optimal energy gain from the substrate to fuel both anabolism and sugar transport, and thus lactate dehydrogenase (LDH) and alcohol dehydrogenase (ADH) should be kept inactive (Figure 2). Indeed, a recent batch-fermentation study has shown that during exponential growth, H_2_, CO_2 _and acetate are the only fermentation products in *C. saccharolyticus *[47]. In this growth phase, neither ethanol nor lactate was formed, although both LDH and ADH activities were detected in crude cell extracts at high levels. However, as the growth rate decreased during transition to the stationary phase, the metabolism was partly directed towards lactate formation. Similar growth phase-related metabolic shifts have been observed in *Cl. cellulolyticum *[48]. Studying the *in vitro *kinetics of LDH activity in *C. saccharolyticus *confirmed the observations of the batch experiments. It revealed that LDH activity in *C. saccharolyticus *is strongly regulated by the levels of the energy carriers PPi and ATP, in addition to the NADH/NAD ratio (Figure 2) [47]. When the cells are growing at the maximum specific growth rate, PPi levels are high and ATP levels are low, keeping LDH inactive and its affinity for NADH low. It further assures that the catabolic flux is directed to acetate and H_2 _(Figure 2). However, as soon as the anabolic activity declines, the PPi/ATP ratio drops by an order of magnitude [71], which results in an increase in LDH activity as well as its affinity for NADH and hence lactate starts being formed [47].

Due to the strong control of *C. saccharolyticus *LDH activity at enzyme level, lactate is formed only in special cases, i.e., at elevated NADH/NAD ratios and at higher ATP levels. High NADH/NAD ratios also lead to inhibition of GAPDH activity and consequently the glycolytic flux [16,47]. Since LDH activity secures the continuation of the glycolytic flux, through diminishing the NADH/NAD ratio, the enzyme can be considered essential for balanced growth in *C. saccharolyticus*. It thus might be questioned whether deleting the *ldh *gene would improve H_2 _yields during sugar fermentation. It might be a better approach to control lactate production through maintaining process conditions that promote exponential growth to keep high cellular PPi levels [47]. It is worth noting that deletion of the *ldh *gene in *E. coli *only slightly increased H_2 _yield and productivities [79].

Ethanol is another reduced end product that is formed during growth of *C. saccharolyticus*, albeit in very low quantities (Figure 2; [13,42]). Based on sequence similarities to the characterized ADH in *T. ethanolicus *[80], ADH in *C. saccharolyticus *possesses a NADPH-binding domain instead of a NADH-binding domain (Figure 2) [26]. This observation is supported by a preliminary kinetic study showing that the affinity of the ADH of *C. saccharolyticus *for NADPH is higher than for NADH [16], indicating that ethanol and H_2 _formation do not compete for the same reduced cofactor.

^13^C-NMR analysis of intact cells has also revealed that *C. saccharolyticus *is capable of reducing either pyruvate or PEP to succinate (Figure 2) in the reductive branch of its incomplete TCA cycle. However, the succinate yield is significantly lower than that of acetate or lactate under regular growth conditions and might only operate under combined elevated *P*_H2 _and *P*_CO2 _levels [31].

Understanding the regulation of the key glycolytic enzyme GAPDH in *C. saccharolyticus *may also contribute to a better understanding of the organism's ability to tolerate elevated *P*_H2_. In general, GAPDH is strongly regulated by the NADH/NAD ratio [81], and GAPDH in *C. saccharolyticus *proved to be no exception [16]. However, the enzyme in *C. saccharolyticus *is more resistant to increased NADH levels than GAPDH in most other related bacteria. The NADH concentration required for 50% inhibition of the enzyme in *C. saccharolyticus *was 0.03 mM, as compared to 0.01 mM in *T. thermohydrosulfuricus *(formerly known as *Cl. thermohydrosulfuricum*) [58] and *Cl. acetobutylicum *[77]. This observation would in theory have a significant impact on the organism's tolerance to H_2_, i.e., if GAPDH was less inhibited by NADH, higher NADH/NAD ratios could be tolerated in the cell without affecting the glycolytic flux. As discussed above, this would also render the hydrogenase reaction more energetically favourable (Eq. 1) [10]. Consistently, the ethanol-adapted *T. thermohydrosulfuricus *strain possesses a GAPDH that tolerates approximately twice the amount of NADH and is more tolerant to H_2 _than the wild-type strain [58]. Likewise, the NADH/NAD ratio in *Cl. cellulyticum *is significantly higher than in *C. saccharolyticus*, which is possibly due to a more relaxed inhibition of its GAPDH by NADH (50% inhibition at 0.1 mM NADH) [42,43].

## 7. Challenges for optimizing hydrogen production by *C. saccharolyticus*

*C. saccharolyticus *has several desirable characteristics needed for a H_2 _cell factory, such as its high H_2 _yields on a wide spectrum of carbon sources, its relatively high tolerance to elevated *P*_H2_, and its inability to reduce S^0^, which therefore does not interfere with its H_2 _production capabilities. However, the organism also lacks several properties, which should be considered before it can be used industrially.

### 7.1 Sensitivity to high osmolalities

Primarily, the sensitivity of *C. saccharolyticus *to increased osmotic pressures is a major drawback that should be addressed. The critical concentration of salts and solutes at which the growth of the organism ceases completely was estimated to be 400-425 mM [30]. Osmolalities above 0.218 osm/kg H_2_O were found to induce cell lysis in *C. saccharolyticus*, as indicated by increased protein and DNA concentrations in the culture supernatant [31]. This low osmotolerance imposes limitations on sparging the culture with CO_2 _and on the maximum sugar concentration in the medium [31], with the latter obviously having a negative effect on H_2 _productivity [30]. On the other hand, the hyperthermophiles *Tm. neapolitana *and *P. furiosus *can grow optimally in the presence of 0.46 M NaCl and 0.5 M NaCl, respectively [82,83]. These salt concentrations correspond to an additional 0.92 and 1 osm/kg H_2_O, respectively, in the culture media, demonstrating that these two hyperthermophiles are better protected against osmotic stress than *C. saccharolyticus*. This higher osmotic tolerance is due to their ability to produce compatible solutes, or osmoprotectants, such as β-mannosylglycerate, di-myo-insitol phosphate and glutamate [82,83]. It remains to be investigated whether *C. saccharolyticus *is also able to efficiently synthesize similar solutes when exposed to osmotic stress. The low salt concentration in its natural habitat [28] may have resulted in the loss of any capability to survive in an unfavourable osmotic environment [82]. However, the presence of biosynthetic genes for some osmoprotectants, such as proline and glutamate, could open the way for directed evolution to render *C. saccharolyticus *more osmotolerant [31].

### 7.2 Low productivities

The low cell density of cultures of *C. saccharolyticus *and other thermophiles, is another major drawback for industrial-scale application. It results in a low volumetric H_2 _productivity [83], unless dialysis or cell-recycling methods are used to increase the cell mass [84]. Especially dialysis was found to increase the cell density of *Pyrococcus furiosus *by an order of magnitude, indicating that the presence of metabolic products prevents the increase in cell densities. In continuous cultures of *C. saccharolyticus*, the volumetric productivity could be enhanced by increasing the dilution rates, but this comes at the expense of the H_2 _yield [13,85]. The decrease in yield could be related to increased dissolved H_2 _concentrations resulting from higher H_2 _productivities[16]. This can inhibit the hydrogenase activity and ultimately results in a metabolic shift to lactate. The same trade off between yield and productivity was also observed with other microorganisms. For instance, *E. coli *produces much lower H_2 _yields but its volumetric productivity is almost 100-fold higher than in *C. saccharolyticus *[13,86]. The cell density is about 200 times higher for *E. coli *[85] than for *C. saccharolyticus *[14], which contributes considerably to the higher volumetric productivity. The specific H_2 _productivity of *E. coli*, however, is low, which could be an undesirable consequence of the higher cell number, i.e., it enhances the H_2 _mass-transfer resistance [85].

### 7.3 Biofilm formation and reactor design

Since *C. saccharolyticus *exhibits a relatively high specific H_2 _productivity, its volumetric production rate can potentially be enhanced through increasing its cell density [83] by, for example, inducing biofilm formation. The reactor configuration is an important factor in enhancing biofilm formation. So far, most research on *C. saccharolyticus *has been carried out in continuously stirred tank reactors (CSTR) [2,13,14,85], which do not allow for efficient biomass retention. Alternative reactors that enhance biofilm formation include trickling-bed bioreactors [11] and fluidized-bed systems with granules [86]. Trickling-bed bioreactors promote biofilm growth by means of packing material with a high surface area within the trickling bed. The liquid passes continuously through the filter, such that it guarantees a good liquid-gas exchange. In the fluidized-bed system, the biofilm is also formed as granules. This reactor system increases the turbulence, thus providing an enhanced mass transfer, which potentially also could decrease the dissolved H_2 _concentration, and hence, improve H_2 _yields. It should be noted that the biofilm mode of growth also offers the surface-attached and matrix-encased bacteria a means of protection against several stressful environmental conditions [87], which could benefit an organism like *C. saccharolyticus *in tolerating higher osmotic pressures. Recently, another *Caldicellulosiruptor *sp., viz. *Caldicellulosiruptor owensensis*, was shown capable of biofilm formation in both trickling-bed and fluidized-bed reactors at 70°C [88]. Moreover, the organism retained its ability to grow and produce H_2 _in the fluidized-bed system even in complete absence of gas sparging.

## 8. Conclusions

This review article has focused on *C. saccharolyticus *as having several desirable characteristics for efficient H_2 _production. This is not only due to the high growth temperature of this organism, but there are strong indications that its metabolic constitution is adapted to efficiently extract energy from carbohydrates, thereby allowing the production of H_2 _at yields close to the Thauer limit [14]. The energy metabolism of *C. saccharolyticus*, based on maximum ATP production per sugar unit and the use of PPi as an alternative energy carrier, might be correlated with the vast number of ABC transporters it employs. The efficiency of the energy metabolism is further illustrated by the modulation of LDH activity in *C. saccharolyticus *through the levels of the energy carriers ATP and PPi [47]; an active LDH will significantly lower the ATP production flux and thus the growth and H_2 _production rates, in addition to draining the electrons required for H_2 _production.

*C. saccharolyticus *can simultaneously co-utilize a wide variety of different sugars [24] and produces efficient extracellular hydrolytic enzymes, including cellulases [21]. It has a relatively high tolerance to partial hydrogen pressures that might be related to its inability to reduce S^0^.

The set of capabilities of *C. saccharolyticus *described above is the foundation to exploit this organism in a commercial process using various raw materials [89]. However, for obvious economical reasons the performance of *C. saccharolyticus *needs to be further improved, i.e., both high H_2 _yields and productivities should be obtained at relatively high *P*_H2 _[6].

Several metabolic engineering strategies can be foreseen to improve H_2 _yields of *C. saccharolyticus *beyond 3.5 mol/mol hexose, without the need for gas sparging. First, the NADH-dependent GAPDH in *C. saccharolyticus *could be replaced with an Fd-dependent GAPOR, similar to the one in *P. furiosus *[54] making the corresponding H_2_-generation reaction spontaneous even at elevated *P*_H2 _[9,36]. Second, introducing the genes for the oxidative part of the PPP supplemented with an NADPH-dependent hydrogenase could allow the oxidation of one mole of hexose to one mole of acetate and four moles of CO_2 _[14,90]. This would in theory yield 8 mol H_2_/mol hexose, but would not help overcome the thermodynamic barrier.

*C. saccharolyticus *exhibits a considerably high specific H_2 _production rate compared to other H_2 _producers [83], but poor volumetric productivities are obtained since its maximum cell densities is in the range of 10^8^-10^9 ^cells/mL [15]. Strategies to increase the cell density by at least an order of magnitude would be through inducing biofilm formation and/or increasing the substrate concentration. The latter requires strains with at least 5-fold increased osmotolerance, which might be obtained through evolutionary engineering. It is noteworthy that *C. saccharolyticus *possesses all the essential genes necessary for biosynthesis of compatible solutes, such as glutamate and proline [31].

As a technical approach, oversaturation of H_2 _in the liquid phase should be avoided to stabilize the fermentation process. This can be solved by smart reactor design and process configuration. Different membrane reactors have been developed, which are capable of reducing dissolved H_2 _concentrations and have a positive effect on H_2 _yields [91-94]. Yet, it remains to be investigated how *C. saccharolyticus *would perform in these reactor systems.

All in all, it is vital to understand the physiology of H_2 _production, which can be facilitated through applying genetic engineering and state-of-the-art 'omics' tools. However, so far, genetic protocols have been developed only for a few thermophilic hydrogen producers [95-98], but not yet for *C. saccharolyticus*. Omics-based technologies are increasingly applied for studying thermophilic H_2 _producers [25,99,100], including *C. saccharolyticus *[24,25]. Together with genome-wide metabolic models, now being developed for some thermophiles [101,102], the systems biology approach can eventually be applied to accelerate our understanding of the physiology of *C. saccharolyticus *and other thermophiles to increase their potential for process applications.

The insights in the physiology of *C. saccharolyticus*, as covered in this review, should contribute to better strategies for the isolation and/or engineering of future superior H_2 _producers. Creating a superior H_2 _cell factory is a quintessential step towards a sustainable biological H_2 _production.

## 9. Competing interests

The authors declare that they have no competing interests.

## 10. Authors' contributions

KW conceived of the study, designed and drafted the manuscript. AAZ contributed to the design and content of the review article and helped to draft the manuscript. EWJN helped to draft the manuscript. AAZ and EWJN critically revised and commented the manuscript.

All authors read and approved the final manuscript.
